# *FLO1*, *FLO5* and *FLO11* Flocculation Gene Expression Impacts *Saccharomyces cerevisiae* Attachment to *Penicillium chrysogenum* in a Co-immobilization Technique

**DOI:** 10.3389/fmicb.2018.02586

**Published:** 2018-10-31

**Authors:** Jaime Moreno-García, Francisco José Martín-García, Minami Ogawa, Teresa García-Martínez, Juan Moreno, Juan C. Mauricio, Linda F. Bisson

**Affiliations:** ^1^Department of Microbiology, University of Córdoba, Córdoba, Spain; ^2^Department of Viticulture and Enology, University of California, Davis, Davis, CA, United States; ^3^Department of Chemistry, Umeå University, Umeå, Sweden; ^4^Department of Agricultural Chemistry, University of Córdoba, Córdoba, Spain

**Keywords:** *FLO* gene, *Penicillium chrysogenum*, co-immobilization, flocculation, *Saccharomyces cerevisiae*

## Abstract

A reoccurring flaw of most yeast immobilization systems that limits the potential of the technique is leakage of the cells from the matrix. Leakage may be due to weakly adherent cells, deterioration of the matrix, or to new growth and loss of non-adherent daughter cells. Yeast biocapsules are a spontaneous, cost effective system of immobilization whereby *Saccharomyces cerevisiae* cells are attached to the hyphae of *Penicillium chrysogenum*, creating hollow spheres that allow recovery and reutilization. This attachment is based on naturally occurring adherent properties of the yeast cell surface. We hypothesized that proteins associated with flocculation might play a role in adherence to fungal hyphae. To test this hypothesis, yeast strains with overexpressed and deleted flocculation genes (*FLO1*, *FLO5*, and *FLO11*) were evaluated for biocapsule formation to observe the impact of gene expression on biocapsule diameter, number, volume, dry mass, and percent immobilized versus non-immobilized cells. Overexpression of all three genes enhanced immobilization and resulted in larger diameter biocapsules. In particular, overexpression of *FLO11* resulted in a five fold increase of absorbed cells versus the wild type isogenic strain. In addition, deletion of *FLO1* and *FLO11* significantly decreased the number of immobilized yeast cells compared to the wild type BY4742. These results confirm the role of natural adherent properties of yeast cells in attachment to fungal hyphae and offer the potential to create strongly adherent cells that will produce adherent progeny thereby reducing the potential for cell leakage from the matrix.

## Introduction

Yeast immobilization provides versatile advantages in industrial fermentation processes such as beer, wine, and biofuel production (reviewed in Moreno-García et al., 2018a). This methodology aims to confine active yeasts cells to a particular domain, thereby concentrating cell population and increasing cell density. The aggregation of cells makes it possible to recover and reutilize the immobilized yeast, allows better control or stability of the yeast strain, protects the yeast against shear forces, and facilitates production and enrichment of certain metabolites (Williams and Munnecke, 1981; Groboillot et al., 1994; Sakurai et al., 2000; Kourkoutas et al., 2004; Baptista et al., 2006; Nedović et al., 2015). Although many benefits have been defined, there are drawbacks that limit the implementation of immobilization systems at industrial scales. Among the most hindering are the problem of cell leaking, or detachment of yeast cell from its support, and the high investment required to integrate these technologies into fermentative practices (Tanaka et al., 1989).

Factors enhancing yeast cell immobilization have been investigated. Studies have utilized chemical pretreatment of the carrier to increase cell loading on the support (Scott and O’Reilly, 1996; Kilonzo et al., 2011; White and Walker, 2011; Lee et al., 2012). However, pretreatment can often times be a costly solution and result in inhibitory by-products (Tomás-Pejó et al., 2009; Behera et al., 2014; Jönsson and Martín, 2016) and if cell division occurs, newly produced cells may not attach. An alternative to these methods is the utilization of “yeast biocapsules,” a spontaneous immobilization system that utilizes the hyphae of a filamentous fungus, *P. chrysogenum*, as a support for yeast cells attachment and that can easily be retrieved for subsequent reuse in fermentation. This biological mechanism is attractive for several reasons (i) being completely natural, (ii) the possibility to genetically engineer both the yeast and the filamentous fungus to improve immobilization, (iii) potential attachment of any newly formed daughter cells using the same natural process as the attached parental cells, and (iv) low investment. In addition, yeast biocapsules have been utilized successfully in production of sparkling wine and natural sweet wine as well as for bioethanol from molasses (Peinado et al., 2005, 2006; García-Martínez et al., 2012, 2013, 2015; López de Lerma et al., 2012, 2018; Puig-Pujol et al., 2013).

Since attachment processes in biocapsule formation rely exclusively on natural adherent properties of the cells, we examined the effect of loss or increased expression of known genes involved in yeast flocculation. Three genes which encode cell wall glycoproteins, *FLO1*, *FLO5*, and *FLO11*; are associated with co-flocculation, defined as the non-sexual aggregation between single-celled organisms of different species (Rossouw et al., 2015). These three genes in addition to *FLO9* have been shown to play a role in biofilm formation on plastic surfaces (Yang et al., 2018). Since to date these genes encode the major cell surface factors documented as functional in cell adhesion in yeasts (SGD, 2018), we speculated that they may also play a role in the *S. cerevisiae*–*P. chrysogenum* attachment and overexpression might consequently improve immobilization. Similarly, deletion of these genes was expected to decrease adherence to the hyphae if flocculation gene products play a role in attachment. Thus, to effectively study the impact of co-flocculating yeast genes expression on biocapsules, we used overexpressed and null mutant strains of *FLO1*, *FLO5*, and *FLO11* and analyzed biocapsule features such as number, diameter, volume, and dry weight, as well as number of cells that were immobilized and not immobilized in the biocapsules.

## Materials and Methods

### Microorganisms and Growth Media

The laboratory strains overexpressing flocculation genes *FLO1*, *FLO5*, and *FLO11* (*S. cerevisiae* FY23 genetic background) were kindly provided by the Institute for Wine Biotechnology [Stellenbosch University, South Africa (SU)] designed by Govender et al. (2008). Null mutants Δflo1 and Δflo11 (*S. cerevisiae* BY4742 genetic background) together with FY23 and BY4742 wild types were obtained from Horizontal Discovery (Table 1). Deletion of *FLO5* in BY4742 results non-viable cells (SGD, 2018) and thus loss of this gene could not be evaluated. Glycerol yeast cultures were streaked out on YPD agar.

**Table 1:** Overexpressed and null *Saccharomyces cerevisiae* mutant strains.

Strain	Genotype	Origin
FY23	*MAT* a *leu2Δ1 trp1Δ63 ura3-52*	Open Biosystems^∗^
BY4742	*MATα his3Δ1 leu2Δ0 lys2Δ0 ura3Δ0*	Open Biosystems^∗^
**Overexpression**
FY23-F1A	*MAT* a *leu2 trp1 ura3 flo8*-*1 FLO1::SMR1-ADH2*	Govender et al., 2008
FY23-F1H	*MAT* a *leu2 trp1 ura3 flo8*-*1 FLO1::SMR1-HSP30*	Govender et al., 2008
FY23-F5A	*MAT* a *leu2 trp1 ura3 flo8* -*1FLO5::SMR1-ADH2*	Govender et al., 2008
FY23-F5H	*MAT* a *leu2 trp1 ura3 flo8*-*1 FLO5::SMR1-HSP30*	Govender et al., 2008
FY23-F11A	*MAT* a *leu2 trp1 ura3 flo8*-*1 FLO11::SMR1-ADH2*	Govender et al., 2008
FY23-F11H	*MAT* a *leu2 trp1 ura3 flo8*-*1 FLO11::SMR1-HSP30*	Govender et al., 2008
**Null**
BY4742 Δflo1	*flo1Δ::KanMX4*	Open Biosystems^∗^
BY4742 Δflo11	*flo11Δ::KanMX4*	Open Biosystems^∗^

*^∗^Strains from open biosystems as part of Dharmacon, a Horizon Discovery Group Co.*

For overexpressed strains, native promotors *FLO1p*, *FLO5p*, and *FLO11p* were replaced with inducible promoters *ADH2p* and *HSP30p* in *S. cerevisiae* FY23 genetic background. The *ADH2* promoter is glucose repressible and known to become de-repressed during the transition to growth on ethanol (Price et al., 1990; Gancedo, 1998; Noronha et al., 1998). The *HSP30* promoter is induced during stationary phase of growth after depletion of glucose from the medium and by other stress factors such as heat shock and sudden exposure to ethanol or sorbate (Régnacq and Boucherie, 1993; Piper et al., 1994; Riou et al., 1997; Seymour and Piper, 1999; Donalies and Stahl, 2001). For biocapsule production, yeast cells were pregrown in rich medium with glycerol as carbon and energy source to stationary phase, a condition that will lead to expression from both promoters. The medium used for immobilization in this work does not contain glucose and cells enter stationary phase during the co-cultivation process, which should sustain activation of both promoters.

Although BY4742 and FY23 are laboratory strain lines derived independently from S288c, they were treated statistically as separate strain genetic backgrounds as they are not genetically identical. Progeny strains, null or overexpression derivatives, were only compared to their respective parental strain as control.

All yeast strains were co-immobilized with *P. chrysogenum* H3, a filamentous fungus strain from the Department of Microbiology (University of Córdoba, Spain) collection (García-Martínez et al., 2011). *P. chrysogenum* H3 was pre-cultured in a sporulation medium (SM) consisting of 1.7% corn meal agar, 0.1% yeast extract, 0.2% glucose, and 2% agar for 7 days at 28°C.

### Biocapsule Formation and Measurement of Biocapsule Parameters

Yeast cells were pre-grown in YP + 3% glycerol medium (175 rpm, 28°C) for 3 days. Next, 150 mL biocapsule formation medium (BFM) in 250 mL flask was inoculated to reach a final concentration of 4 × 10^6^ yeast cells/mL in the presence of a total of 4 × 10^6^ *P. chrysogenum* spores. Biocapsule formation was performed in triplicate. BFM consists of 0.67% yeast nitrogen base medium without amino acids (Difco) and 5 g/L gluconic acid as a carbon source. The BFM was buffered to pH 7 with sodium and potassium phosphate. Flasks were shaken at 175 rpm at 28°C for 6 days. This procedure is the same as previously published protocols (Peinado et al., 2004; García-Martínez et al., 2011).

After 6 days, biocapsules were removed from the BFM and washed with distilled water to measure parameters such as immobilized and non-immobilized yeast cells, immobilized yeast cells per biocapsule, immobilized yeast cell per mg of biocapsule and number, diameter, total volume, and dry weight of biocapsules. After the biocapsules removal, the medium was used to count free or non-immobilized yeast cells to further calculate immobilized yeast cells percentages. Number of biocapsules per flask were counted and the size of each biocapsule diameter and volume of the sum of all biocapsules in each flask measured. Immobilized yeast cells were quantified by taking ten biocapsules per flask and “disrupting” biocapsules with NaCl (100 mM), grinding them with a mortar and pestle for 2 min, and vortexing for 20 s in order to separate yeast cells from fungal hyphae. To clarify the sample from the fungus hyphae so that the yeast cells could be quantified, several successive differential filtrations were performed following Moreno-García et al. (2018b). Yeast cells were quantified by analyzing the filtered samples under a microscope and counting cells on a Hemocytometer at 40× objective and finally the number was normalized to the total number of biocapsules in each replicate. Free yeasts were also quantified by Hemocytometer. The remainder of biocapsules from each flask were analyzed for dry weight by desiccating the biocapsules in 105°C constant temperature overnight and weighing them the next day.

### Statistical Analysis

**FIGURE 1:**
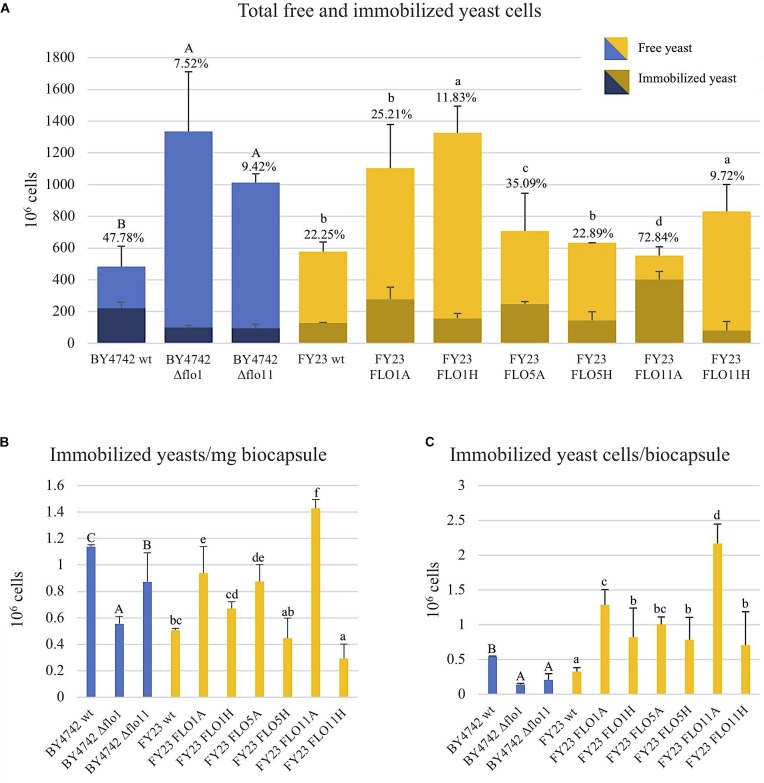
Yeast immobilization potential of null mutant strains versus BY4742 wt (blue) and overexpressed mutant strains versus FY23 wt (yellow). A and H after each strain name represent *ADH2* and *HSP30* promoters, respectively. **(A)** Yeast cells immobilized (dark color areas) and yeast cells non-immobilized (light color areas) considering each replica. Percentages of yeast cell immobilized out of total population are indicated for biocapsules made with each yeast strain. **(B)** Immobilized yeast cells per mg of biocapsule. **(C)** Immobilized yeast cells per biocapsule. In **(A–C)**, homogenous groups are represented with alphabets where capital letters compare within biocapsules made with the null mutant and wild type, and lower-case letters compare biocapsules made with overexpressed mutants and wild type.

**FIGURE 2:**
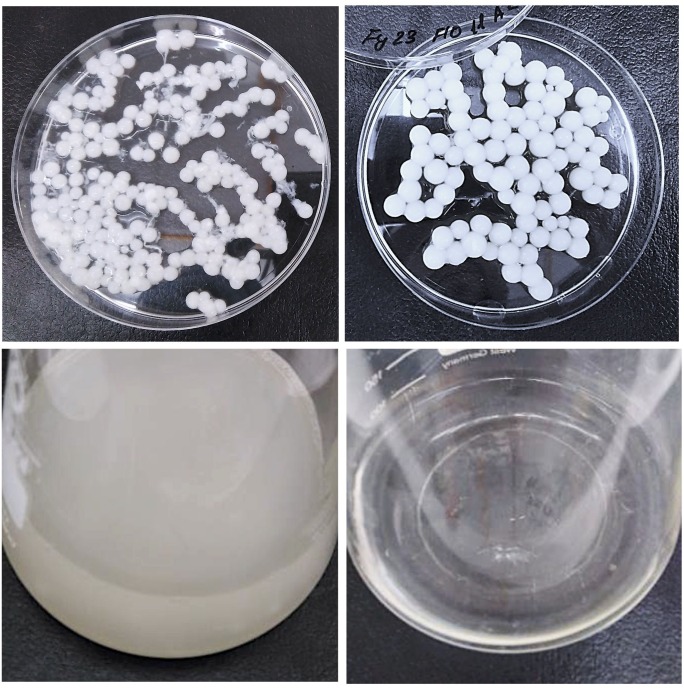
FY23 wt (left) and FY23 FLO11A (right) biocapsules (up) and non-immobilized yeast cells (down) after biocapsules were retrieved.

**FIGURE 3:**
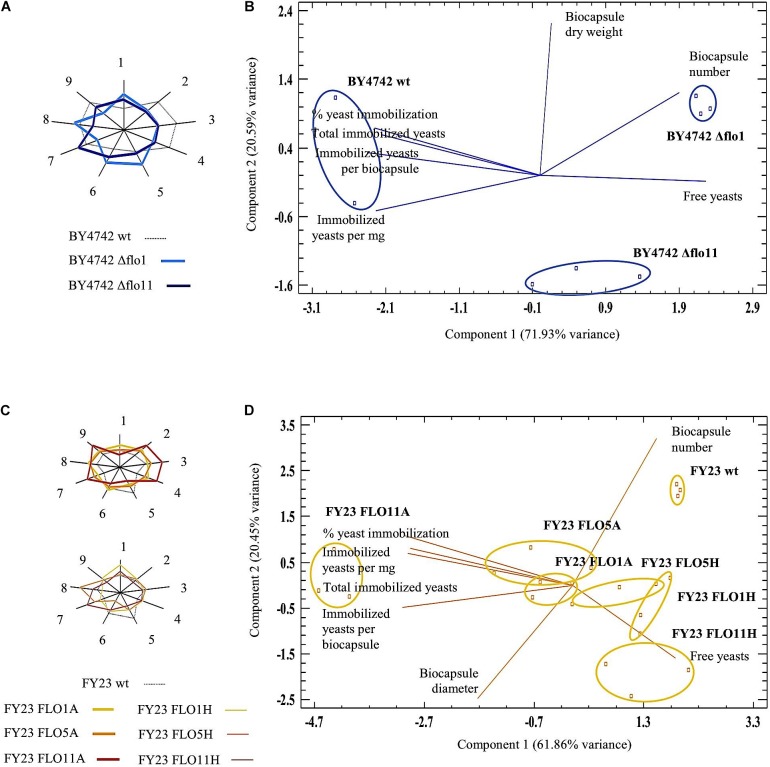
Multiple Variable Analysis (MVA) sunray plots and Principal Component Analysis (PCA) for biocapsules made with null mutants **(A,B)** and overexpressed mutants **(C,D)**, respectively. In **(A,C)**, each ray in the non-agon corresponds to one parameter: (1) free yeasts; (2) total immobilized yeasts; (3) % yeast immobilization; (4) immobilized yeast per biocapsule; (5) biocapsule number; (6) biocapsule total volume; (7) biocapsule diameter; (8) biocapsule dry weight; (9) immobilized yeast per mg biocapsule. The end of the ray is the mean value plus three standard deviations and the center the mean minus three standard deviations. **(A,B)** compare parameters between null strains and BY4742 wt; **(C,D)** compare parameters of overexpressed strains and FY23 wt. In **(B,D)**, Principal Components Analyses Biplot were carried out with seven variables selected by their discrimination power among the yeast strains studied.

All obtained results were analyzed with a statistical analysis software Statgraphics Centurion XVI (Manugistics, Inc., Rockville, MD, United States) (Figures 1–3, 5–8). Homogenous groups are represented by different letters indicating groups with significant differences at 0.05 level according to the *F*-test (Figures 1, 5–8). The data was subjected to Principal Component Analysis (PCA) and Multiple Variable Analysis (MVA) (Figure 3).

## Results

The impact of the expression of yeast gene*s FLO1*, *FLO5*, and *FLO11* on attachment of yeast cells to the fungal hyphae walls was studied using genetically altered yeasts compared to the unaltered parental strain. Biocapsule number, diameter, volume, and dry weight as well as the number of cells that were immobilized and not immobilized by the biocapsules were assessed.

### *FLO1*, *FLO5*, and *FLO11* Overexpression Increases Immobilization Ability

Immobilization ability of control and *FLO* gene overexpressing strains was assessed by comparing the number of yeast cells immobilized in the total biocapsules per replica to the total number of free or non-immobilized cells and expressed as a percentage of immobilization (Figure 1A). In the deletion analyses, Δflo1 and Δflo11 significantly decreased the number of immobilized yeast cells compared to the wild type BY4742, dropping to 8–9% immobilization from 48% for the control. The deletant cells grew better under the medium conditions used than the wild type strain, but, in spite of more cells being present, the number of attached cells was reduced. This suggests that for this strain attachment may limit cell reproduction and remove cells from the actively budding population. For the overexpression studies, overexpression of *FLO5* and *FLO11* using the *ADH2* promoter statistically and significantly increased immobilization percentages, rising 35 and 73% respectively, from 22% for the wild type FY23. On the other hand, expression using the *HSP30* promoter significantly decreased immobilization percentages for *FLO1* and *FLO11* as compared to the control. Govender et al. (2008) measured the QRT-PCR expression of *FLO1*, *FLO5*, and *FLO11* transcripts using the native, *ADH2*, and *HSP30* promotors and reported higher expression under inducting condition of absence of glucose and entry into stationary phase for both the *HSP30* and *ADH2* constructs as compared to the native promoter. These authors also observed that those strains with *HSP30* promoter expression displayed lower values for phenotypes that may be related with the fungus-yeast attachment, such as biofilm formation, buoyancy, and hydrophobicity compared to *ADH2*, consistent with the data obtained in our work. The increased immobilization ability with the overexpression of the *FLO5* and *FLO11* genes using the *ADH2* promotor and the decrease in immobilization with the gene deletions *FLO1* and *FLO11* suggests that the co-flocculation gene products are relevant components for yeast cell attachment to the filamentous fungus.

Immobilized yeast cells per mg of biocapsule was calculated by dividing the total number of immobilized cells by total dry weight of biocapsules per sample (Figure 1B). Here, null mutants of *FLO1* and *FLO11* had significantly less cell density than wild type and *FLO1*, *FLO5*, and *FLO11*, when overexpressed with *ADH2* promotor, had higher cell concentration than the wild type. When overexpressed with *HSP30* promotor, *FLO11* resulted in decreased cell density per mass from the control. *FLO1* and *FLO5* expression from the *HSP30* promoter yielded values similar to the control strains.

Immobilization ability was also assessed per yeast biocapsule where the total number of immobilized cells per sample was divided by the total number of biocapsules (Figure 1C). All overexpressed strains outperformed the wildtype, where FLO11A had five fold more immobilized cells per biocapsule compared to wild type. Results could clearly be observed with the naked eye by the apparent decrease of turbidity of the BFM medium (Figure 2). Correspondingly, all null mutations significantly decreased in immobilization ability compared to wild type.

The differences of Δflo1 and Δflo11 from the wild type BY4742 can be visualized in the sunray plots (Figure 3A) where all nine biocapsule parameters are compared. Out of those nine parameters, seven accounted for significant differences and were analyzed by PCA biplot (Figure 3B). Figure 3B shows the distribution of null *FLO* gene samples and the seven biocapsule parameters to each PC plotted in the planes defined by PC1 and PC2 (accounting for 92.52% total variance). The variables “free yeasts” and “biocapsule number” were those that most differentiated the null mutants from the wild type.

For overexpressed strains, FLO11A displays the most distinctive footprinting in the sunray plot and can be defined by the greatest immobilization ability compared all other strains (parameters 2, 3, 4, and 9) (Figure 3C). Also shown in the PCA biplot (Figure 3D), biocapsules made with FLO11A lies furthest left and separate from FY23 wild type and the other overexpressed strains, where “% yeast immobilization,” “immobilized yeasts per mg biocapsule,” “total immobilized yeasts,” and “immobilized yeasts per biocapsule”; are variables that discriminate most.

### Overexpression of *FLO1*, *FLO5*, and *FLO11* Produce Fewer but Larger Biocapsules

**FIGURE 4:**
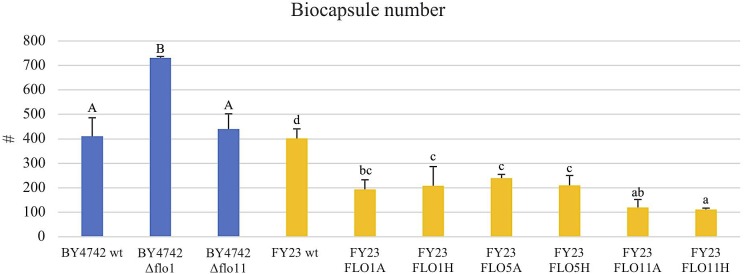
Average of biocapsule number of null mutant strains versus their wild type BY4742 wt (blue) and overexpressed mutant strains versus their wild type FY23 wt (yellow). Homogenous groups are represented with alphabets where capital letters compare within the null mutant and wild type, and lower-case letters compare within overexpressed mutants and wild type.

**FIGURE 5:**
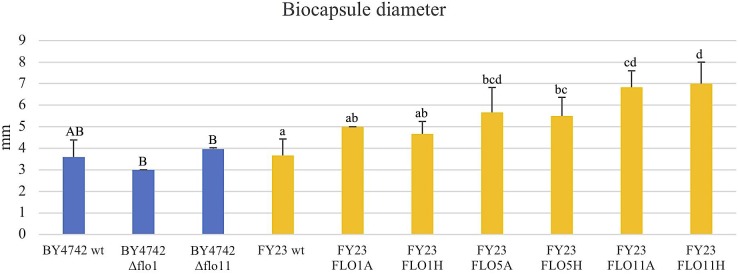
Biocapsule diameter measured in mm of null mutant strains versus their wild type BY4742 wt (blue) and overexpressed mutant strains versus their wild type FY23 wt (yellow). Homogenous groups are represented with alphabets where capital letters compare within the null mutant and wild type, and lower-case letters compare within overexpressed mutants and wild type.

**FIGURE 6:**
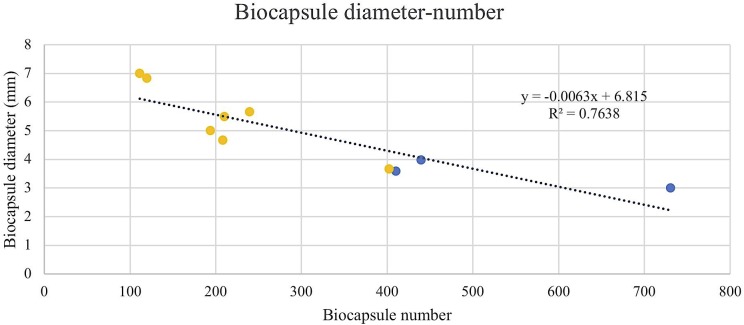
Linear regression of biocapsule diameter and number. Null mutant strains and their wild type BY4742 wt are represented in blue and overexpressed mutant strains and their wild type FY23 wt in yellow.

To assess the Flo protein impact on the physical properties of yeast biocapsules, measurements of the number of biocapsules formed and the average of the diameter of each biocapsule were assessed (Figures 4, 5). The two parameters resulted in a negative linear regression (Figure 6) indicating that those strains with capacity to form larger biocapsules make less biocapsules than those that make smaller biocapsules. In this study, the overexpressed mutants generally yielded larger but less abundant biocapsules while the null mutants were the opposite, smaller, and more abundant biocapsules. In particular, overexpression of *FLO11* using either promoters, resulted in the largest and fewest biocapsules within all the samples. This finding is intriguing because overexpression of *FLO11* from the *HSP30* promoter did not result in a higher percentage of cells binding to the matrix versus the wild type yet yielded biocapsules of similar number and diameter to the overexpression from the *ADH2* promoter. In these strains the level of expression or of growth of the cells impacted biocapsule parameters without enhancing binding of the yeast to the capsule. These trends further suggest that co-flocculation proteins may possess properties that influence the structure of the biocapsules to be larger, especially Flo11p. Intuitively speaking, larger biocapsules are able to entrap more cells per biocapsule as reflected in Figure 1C. Thus, the properties of the Flo proteins could potentially help define the structure of the biocapsules themselves, resulting in an increase in size and further increase number of cells attached per biocapsule.

### Mass and Volume Are More Strain Dependent Than *FLO* Gene Dependent

**FIGURE 7:**
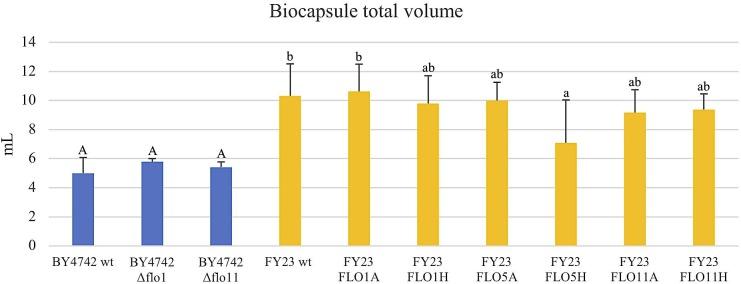
Total volume of the biocapsules per replica of null mutants strains versus their wild type BY4742 wt (in blue) and overexpressed mutant strains versus their wild type FY23 wt (yellow). Homogenous groups are represented with alphabets where capital letters compare within the null mutant and wild type, and lower-case letters compare within overexpressed mutants and wild type.

**FIGURE 8:**
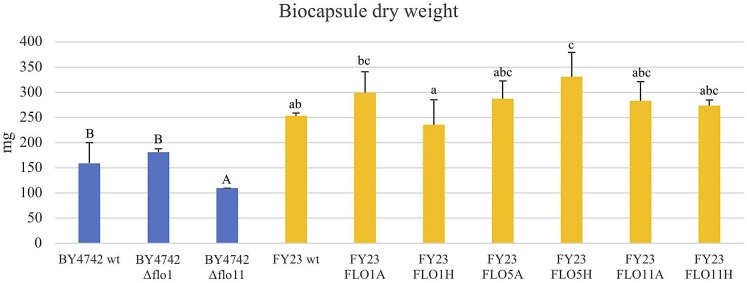
Dry weight of the biocapsules of null mutants strains versus their wild type BY4742 wt (in blue) and overexpressed mutant strains versus their wild type FY23 wt (yellow). Homogenous groups are represented with alphabets where capital letters compare within the null mutant and wild type, and lower-case letters compare within overexpressed mutants and wild type.

The total dry weight and total volume of the yeast biocapsules did not significantly differ with overexpression or deletion of the *FLO* genes compared to the respective wild type controls with the exceptions of FLO5H and Δflo11 (for dry weight only) (Figures 7, 8). A difference between the two strains BY4742 and FY23 is apparent, all biocapsule samples of strain FY23 and its derivatives were greater in mass and volume compared to those for BY4742 and its derivatives. When compared to the dependence of biocapsule number and diameter on yeast strain, the constancy of the total dry weight suggests that the total mass of biocapsules is associated with the fungus mass but the parameters of the individual capsules, size, and number, are influenced by yeast strain. These data further suggest that factors affecting the mass and volume of the yeast biocapsules may be more strain dependent than attachment dependent. Biocapsule number and diameter are indistinguishable for *FLO11* when expressed from either the *ADH2* or *HSP30* promoters, but the level of attachment of the cells varies dramatically (Figure 1C), suggesting that strain parameters but not the physical action of attachment dictate biocapsule number and diameter. Moreno-García et al. (2018b) supports this result with an earlier experiment and found that mass and volume of yeast biocapsules are highly strain dependent.

## Discussion

Yeast immobilization, though advantageous, chronically suffers from the detachment of cells from the carrier. In this study, yeast attachment in biocapsules using genetically altered yeasts, with overexpressed co-flocculating genes *FLO1*, *FLO5*, and *FLO11* and deleted *FLO1* and *FLO11* was evaluated. The assessment of the impact of deletion of *FLO5* was not able to be undertaken as this mutant is not viable. After various analyses of different biocapsule parameters, we found that overexpression of these genes increases immobilization ability and cell attachment using the *ADH2* promotor while the deletion of the genes caused a decrease in these parameters. Attachment of yeast cells to each other is mediated by flocculins, transcribed by the *FLO* genes, which confer adhesion in cell–cell or cell-substrate interactions (Bony et al., 1998; Fichtner et al., 2007). Flo1p and Flo5p are flocculins that selectively bind to α-mannose residues of the surface of neighboring cells (Miki et al., 1982; Teunissen and Steensma, 1995; Kobayashi et al., 1998). *P. chrysogenum* cell wall is composed of some quantities of mannose (Applegarth, 1967). Our data suggest these yeast flocculins can bind to these residues and thus increase cell adherence in biocapsule formation.

Flo11p when overexpressed from the *ADH2* promoter showed a much stronger impact on biocapsule adhesion than the other two proteins. Flo11p differs from Flo1p and Flo5p in that in addition to flocculation, it is involved in processes such as cell-substrate adhesion and it is an essential protein for biofilm formation and haploid invasive growth (Reynolds and Fink, 2001; Fichtner et al., 2007; Van Mulders et al., 2009). In this study, overexpression of *FLO11* immobilized the most total cells, had the highest cell per mg biocapsule and most cells per biocapsule. Interestingly, sequence comparisons of flor strain genomes to that of the laboratory wild type S288C showed modifications of the promoter region of *FLO11* that would lead to greater expression of this gene but observed changes that would decrease expression of other flocculin genes (Eldarov et al., 2018).

The robust strength of attachment of *FLO11* compared to the other flocculation genes could be due to a combination of the additional biological properties that *FLO11* confers. Cell-substrate adhesion and biofilm formation are biological processes enabling microorganisms to adhere to solid surfaces or aggregate in air-liquid interfaces, respectively, (Fidalgo et al., 2008; Váchová et al., 2011). Hydrophobic forces of the surfaces of yeast cells are believed to drive these interactions (Van Mulders et al., 2009). Govender et al. (2008) found that cell surface hydrophobicity was the highest in strains overexpressing *FLO11* when studying the optimization of flocculent behavior by overexpressing *FLO1*, *FLO5*, and *FLO11*. It may be that in the case of the biocapsules, hydrophobic forces of Flo11p causes yeast cells to repulse from the liquid media and attract instead to the hydrophobic fungal spores and/or hyphae (cell-substrate adhesion) and then form biofilm within the hyphae matrix leading to stronger attachment (Zhang and Zhang, 2016). Consistent with this observation, in a previous study (Moreno-García et al., 2018b), yeasts that were naturally able to form biofilm also showed higher rates of immobilization in biocapsules when compared with yeasts lacking this ability. Moreover, *FLO11* can induce haploid invasive growth (Lambrechts et al., 1996; Harashima and Heitman, 2002; Kuchin et al., 2002). This biological process is defined as a growth pattern where the cells become elongated and fail to separate after division, resulting in physical penetration of the cells into an agar medium (Van Mulders et al., 2009). In biocapsules, overexpressed *FLO11* strains could potentially invasively grow into the solid fungal hyphae structure in a similar fashion. This strong adhesion allows yeast cells to withstand aggressive forces such as rinsing and physical rubbing (Van Mulders et al., 2009). Such forces will commonly be applied when biocapsules are used in industry scale and to be able to tolerate such conditions would favor their application and improve prevention of cell leaking. The overexpression from the *ADH2* promoter leads to greater biocapsule attachment than overexpression from the *HSP30* promoter may suggest that the density of Flo11 proteins on the cell surface is an important factor in attachment. If too low or too high attachment may not occur. This is consistent with previous observations comparing the impact of Flo11p expression from these same promoters on biofilm formation and decreased biofilm formation seen with expression from *HSP30* (Govender et al., 2008).

The ability of Flo proteins to be able to adhere to neighboring cells or other substrates may also explain the trend of large size but few in number of biocapsules (Figures 4–6). This linear regression matches with that of a previous study by Moreno-García et al. (2018b), where biocapsules made with different wild type yeast strains displayed large-few or small-abundant trends. Overexpressed *FLO11* strains resulted in the largest diameter biocapsules perhaps because Flo11p allows for attachment with a longer retention and more permanency compared to Flo1p and Flo5p which are involved in flocculation only and no other FLO11 phenotypes, where attachment is easily broken by intense agitation.

The co-aggregation of yeast with the *P. chrysogenum* could be a social mechanism as a mean of survival for the *S. cerevisiae*. By adhering to a localized space on and within the hyphae walls of the filamentous fungus, the yeasts can cooperate as a cell community to survive in an environment such as the BFM where there are only carbon sources that are difficult for *S. cerevisiae* to metabolize (i.e., gluconic acid) (Peinado et al., 2003). Perhaps the yeasts may be obtaining sub-products of the gluconic acid metabolism from the fungus which are more accessible to utilize as an energy source and the yeasts are forced to maintain a relationship with the fungus. Furthermore, the matrix of the fungal hyphae acts as an anchoring point for the yeasts as well as a physical shield against stress factors that a free-living single cell cannot tolerate on its own.

This study highlights the impact of the expression of co-flocculating genes *FLO1*, *FLO5*, and *FLO11* on yeast biocapsule formation via genetic engineering. The overexpressed strains using *ADH2* promotor produced biocapsules that were high in immobilization efficiency, larger in individual size but fewer in total number while null mutants had opposite results. In particular, *FLO11* overexpression with *ADH2* promotor significantly outperformed the other co-flocculating genes in all parameters and immobilized 73% cells compared to its wild type that was 22%. In order to assure adhesion of any progeny cells produced during wine fermentation other promoters will need to be considered given the high sugar content of grape juice. *FLO* gene regulation during fermentation is under complex control (Li et al., 2015) and thus multiple targets exist for modulation of flocculin expression.

To further increase adsorption of cells to the filamentous fungus, future studies overexpressing all three concurrently or alternating combination of co-flocculation genes could be utilized. These genomic alterations of the overexpressed strains used in this study can be regarded as self-cloned and are GRAS, or generally recognized as safe (Verstrepen et al., 2003). Such strains have the potential of being purposed for industry, including food/beverage manufacturing practices, and could readily be utilized to upscale to improve production capabilities. Additionally, the fact that the two promoters, *ADH2* and *HSP30*, used in this study activate in glucose lacking conditions means that they can be triggered in fermentation like conditions (e.g., wine, beer, and bioethanol), thus expressing *FLO* genes and boosting yeast cell immobilization in biocapsules. Also, given that biocapsules formed using these well-characterized laboratory strains mimic the diversity in biocapsule parameters seen across wine strains evaluated previously (Moreno-García et al., 2018b) these strains may be used to compare the fermentative capability and stability of larger versus smaller biocapsules.

While studying the molecular mechanism by which yeast and filamentous fungus attach harbors potential solutions for enhancing alternative forms of immobilization systems, it also facilitates perspective into understanding biological social interactions of organisms of differing subphyla. To our knowledge, this is the first time that *S. cerevisiae* co-flocculating genes – up until now only defines adhesion between differing unicellular species – are found to be associated with attachment of unicellular organisms with multicellular organisms. Our data opens perspective on functional capacities of these genes related to interactions within mixed communities beyond the scope of what we currently know which allows associations to be maintained and serve as a strength of the cell community as a whole.

## Author Contributions

JM-G conducted the study and analyzed the experimental data and drafted the manuscript along with MO. FM-G and MO also contributed equally to conducting parts of the study. JM and LB helped analyze and review the experimental data. TG-M, JM, and JCM participated in the selection and interpretation of the statistical analysis applied and in the coordination of the study. All authors reviewed drafts and contributed to writing the manuscript. All authors approved the final version of the manuscript.

## Conflict of Interest Statement

The authors declare that the research was conducted in the absence of any commercial or financial relationships that could be construed as a potential conflict of interest.
